# Effect of Disinfectants on Preventing the Cross-Contamination of Pathogens in Fresh Produce Washing Water

**DOI:** 10.3390/ijerph120808658

**Published:** 2015-07-23

**Authors:** Jennifer L. Banach, Imca Sampers, Sam Van Haute, H.J. (Ine) van der Fels-Klerx

**Affiliations:** 1 RIKILT - Wageningen UR (University and Research Centre), P.O. Box 230, 6700 AE Wageningen, The Netherlands; E-Mail: ine.vanderfels@wur.nl; 2 Laboratory of Food Microbiology and Biotechnology, Department of Industrial Biological Sciences, Faculty of Bioscience Engineering, Ghent University Campus Kortrijk, Graaf Karel de Goedelaan 5, Kortrijk B-8500, Belgium; E-Mails: imca.sampers@ugent.be (I.S.); sam.vanhaute@ugent.be (S.V.H.)

**Keywords:** water disinfection, fresh produce, cross-contamination, chlorine, chlorine dioxide, peracetic acid, ozone, disinfection by-products, water quality

## Abstract

The potential cross-contamination of pathogens between clean and contaminated produce in the washing tank is highly dependent on the water quality. Process wash water disinfectants are applied to maintain the water quality during processing. The review examines the efficacy of process wash water disinfectants during produce processing with the aim to prevent cross-contamination of pathogens. Process wash water disinfection requires short contact times so microorganisms are rapidly inactivated. Free chlorine, chlorine dioxide, ozone, and peracetic acid were considered suitable disinfectants. A disinfectant’s reactivity with the organic matter will determine the disinfectant residual, which is of paramount importance for microbial inactivation and should be monitored *in situ*. Furthermore, the chemical and worker safety, and the legislative framework will determine the suitability of a disinfection technique. Current research often focuses on produce decontamination and to a lesser extent on preventing cross-contamination. Further research on a sanitizer’s efficacy in the washing water is recommended at the laboratory scale, in particular with experimental designs reflecting industrial conditions. Validation on the industrial scale is warranted to better understand the overall effects of a sanitizer.

## 1. Introduction

Microbial food safety and quality issues with leafy vegetables, including the presence of pathogens on leafy greens, have been reported. During the production of fresh-cut vegetables, the washing step has been identified as a potential pathway for dispersion of microorganisms, and more specifically *Escherichia coli* (*E. coli*), to the end product [1]. Although washing with potable water helps to remove microorganisms to a certain extent, sanitizers have also been applied to enhance the disinfection of the produce (*i.e.* decontamination) [2,3]. Nevertheless, the ability to remove naturally present microorganisms from fresh-cut produce is limited (0.5–2.0-log reduction), *i.e.*, some microbial reductions occur, but total reduction is unachievable [4,5,6,7]. These limitations are attributed to microbial attachment to surfaces, including those at crevices or cut edges, or as a result of irregular surface structures. In addition, microorganisms may form biofilms, or become internalized within plant tissues, through stomata, cut surfaces, or other tissue wounds, or during the pre-harvest stage via the root system, although the significance of the latter has yet to be confirmed [1,8,9,10,11]. In short, the disinfectant dose used to avoid cross-contamination is lower compared to the dose needed for microbial inactivation in the fresh produce, thus reducing the formation of disinfection by-products (DBPs). Therefore, sanitizing strategies should focus primarily on preventing cross-contamination in the washing tank rather than on ensuring produce safety as a result of product decontamination. The potential cross-contamination during washing between clean and contaminated produce may be minimized by maintaining the water quality throughout processing [12,13,14], since once the produce becomes contaminated, decontamination of the final product is unlikely to remove attached microorganisms [14,15]. In particular, Gil, Selma, López-Gálvez and Allende [12] have reviewed fresh-cut produce sanitation and wash water disinfection outlining the problems and potential solutions to current applications. These authors highlight that sanitizers are key for hygiene in the fresh-cut produce industry, yet stress that water disinfection should be aimed at preventing cross-contamination between clean and contaminated products. In other words, sanitizers are used to maintain the quality of the washing water despite limited, direct microbial benefits on the produce [12]. Davidson *et al.* [16] have also stressed this argument, stating that the aim of sanitizer application is mainly to lessen the effects of cross-contamination during washing and have concluded that sanitizers are not to be used to guarantee product safety. Such conclusions have also been highlighted by other authors [16,17,18]. 

In the fresh-cut produce industry, water disinfection occurs in washing tanks, or immersion washers, in which the produce are washed [19]; however, water disinfection can also be used to recycle process water (*i.e.* water reconditioning) [20]. Process wash water and water reconditioning can be distinguished based on where and when the disinfection takes place: (i) process wash water disinfection occurs in the washing tank where a disinfectant residual is maintained during washing, *i.e.*, all the water is treated, and (ii) water reconditioning occurs outside the washing tank where only part of the water is treated at a specific time [14,15]. A distinction between these disinfection methods is important when considering the potential for reducing microbial cross-contamination. For example, during water reconditioning, microbial inactivation occurs at another location than the contamination event (*i.e.*, the washing tank), which increases the probability of cross-contamination, such as pathogen point-contaminations [14].

The effect of disinfectants on reducing cross-contamination can be estimated using disinfection kinetics. Disinfection kinetics are based on the disinfectant dose (chemical, irradiation, or ultrasound (US) power consumption) and contact time (concentration × time). Water disinfection treatments demonstrate vastly different kinetic behaviors towards microorganism inactivation, which are dependent on the inherent disinfection efficiency as well as the influence of the physicochemical quality of the water matrix on the disinfectant concentration. The exposure (*i.e.* contact time) is a major limiting factor for process wash water disinfection due to required short contact times (30 seconds up to a few minutes); thus, microorganisms in the wash water must be instantaneously inactivated [14,20]. 

Due to the increasing presence of organic matter in the wash water during a production cycle, the disinfectant dose reduces over time. This reduction demonstrates the premise for a disinfectant residual during washing. Principally, wash water disinfection of process washing water with sanitizers can only function adequately for preventing cross-contamination when the required disinfectant residual is controlled in the washing bath through automated monitoring and dosing of the disinfectant. 

Water quality of the process wash water can only be maintained when disinfection kinetics favor quick, efficient disinfectants. To date, the following chemical disinfectants appear to be appropriate for process wash water disinfection: free chlorine, chlorine dioxide (ClO_2_), ozone (O_3_), and peracetic acid (PAA). This review explores the influences on the disinfection efficacy of these disinfectants in the wash water during produce processing with the aim to prevent cross-contamination of pathogens on the fresh produce via the process wash water. 

## 2. Selection of Wash Water Disinfectants

Besides free chlorine, ClO_2_, O_3_, and PAA, other disinfectants are currently available; however, these options appear less suitable for preventing cross-contamination during fresh produce washing, *i.e.* during process washing [14]. In other words, processing limitations influence the appropriate sanitizer choice for disinfection techniques. The limitations for wash water disinfection are: (i) how the sanitizer can be used in the wash water system, and (ii) the necessary disinfectant residual (which can be based on physicochemical parameters like the organic matter) in order to achieve sufficient disinfection. Specifically, current applications for hydrogen peroxide (H_2_O_2_), organic acids, US, and ultraviolet (UV) irradiation should not be recommended as process wash water disinfectants for fresh produce. 

H_2_O_2_ requires a high residual alongside a high disinfectant demand due to its strong influence with organics in the wash water, and thus rapid consumption and slow disinfection kinetics [21]. Organic acids require high concentrations to be applied, yet despite these concentrations, minimum effective doses for strong organic acids, like acetic or lactic acid, exceed levels that would prevent adverse effects on the sensory quality of produce [7,18,22,23]. Current ultrasonic generating devices are not effective at delivering rapid inactivation of microorganisms. The necessary energy demand is too high for application during process wash water disinfection [24,25,26]. With respect to fresh-cut produce wash water, Gómez-López, *et al.* [27] demonstrated that increasing the chemical oxygen demand (COD) in the wash water did not influence the inactivation of *E. coli* O157:H7. However, current ultrasonication methods require high sonication intensities and long contact times making these methods impractical when preventing microbial cross-contamination in the process wash water [20,28]. UV water disinfection involves circulating a thin layer of water along suspended UV lamps. This type of setup is required since UV transmittance in the water is highly influenced by the presence of organic matter or suspended particles, which can absorb or shield UV rays [14,29]. Evidently, UV application in the fresh produce process wash water would have considerable UV absorbance. Furthermore, traditional washing tank designs prevent the close proximity of all water in the washing tank to the lamps at all times, which would be a prerequisite in order to prevent cross-contamination utilizing this method. Therefore, UV irradiation is *de facto* a reconditioning technique and is not optimal when preventing microbial cross-contamination [14]. 

These aforementioned wash water disinfectants or current water treatment technologies are simply not appropriate for preventing cross-contamination in fresh produce washing operations owing to the need for very high disinfectant residuals (or electrical energy consumption), which is due to the slow microbial inactivation kinetics and/or great interference from organic matter.

## 3. Sanitizer Efficacy

Sanitizer efficacy depends on many parameters such as the disinfectant type, dosage, residual concentration, contact time, temperature, pH, produce to water ratio in the process wash water where the sanitizer is applied, and the extent of organic matter in the washing tank as well as other physicochemical properties of the process wash water [12,15,16].

Gil, Selma, López-Gálvez and Allende [12] note the difficulties in comparing the efficacy of disinfection technologies as several factors in the experimental set-up influence the efficacy. These include the water quality, sanitizers, target microorganisms, inoculation procedure (e.g., spray, submersion, *etc.*), methods of detection, produce, and time interval [12]. In order to evaluate potential disinfection treatments, Van Haute, Sampers, Jacxsens and Uyttendaele [14] have evaluated several water disinfection technologies, aimed at inactivating pathogenic microorganisms, in order to characterize criteria that influence the suitability of a technology and provide a selection tool for fresh-cut produce methods during pre- and post-harvest practices. 

Overall, the process wash water quality is of upmost concern since during processing the composition is perpetually changing and is a cause of concern for potential microbiological contaminations [18,30]. Furthermore, the process wash water quality is influenced by the disinfectant choice [15], while the efficacy of a sanitizer is a function of the disinfectant residual. The efficiency of wash water disinfection is not restricted to issues that affect produce decontamination, but the effectiveness of chemical oxidants is rather hindered by the presence of organic matter in the wash water [14,18,20,21]. In particular, the influence of physicochemical parameters (e.g., organic matter) on the stability of the processing wash water disinfectants can be summarized as a function of decreasing reactivity: O_3_ > HOCl (hypochlorous acid) > ClO_2_ > PAA [14,15,18,31,32]. Thus, when there is a decreasing reactivity with the organic matter, there is a decreasing dose that is necessary to maintain the residual. The general disinfection efficacy of process wash water disinfectants is as follows: O_3_ > HOCl ≈ ClO_2_ > PAA. Since a fast inactivation in the washing tank is of paramount importance, the disinfectant residual is the parameter that can be adjusted. Accordingly, the amount of necessary disinfectant residual is as follows: PAA> HOCl ≈ ClO_2_ > O_3_. In brief, the organic matter in the process wash water has shown to influence the disinfectant demand and dosage in order to maintain a disinfectant residual, of which is important to maintain continuously throughout processing [14,15,27]. 

Furthermore, the target microorganism in question is an important consideration when selecting an appropriate process wash water disinfectant. Whether it be vegetative bacteria, bacterial spores, Gram-positive pathogens (e.g., *Listeria monocytogenes* (*L. monocytogenes*)), Gram-negative pathogens (e.g., *E. coli* O157:H7 or *Salmonella* spp.), viruses (Norovirus or bacteriophages), or protozoa (*Giardia* or *Cryptosporidium*), or even several of these, this choice influences the achievable microbial reduction and disinfection strategy to be applied. Van Haute, Sampers, Jacxsens and Uyttendaele [14] have evaluated the influence of the microorganism type on process wash water disinfectants. The surrogate type investigated (e.g., murine norovirus (MNV) for Norovirus) as well as the inoculum composition, mixed or cocktail strains, also play a role in the efficacy of the applied technology during experiments. In general, the necessary disinfection residuals to inactivate vegetative bacteria in the wash water *versus* on the produce, e.g., for free chlorine, differ as such: residuals of 1–5 mg/L inactivate microorganisms in the wash water (wash water disinfection), whereas residuals of 20–200 mg/L or more are usually applied for inactivation of microorganisms on fresh produce (*i.e.* decontamination) [3,7,13]. The differences in the doses required to avoid cross-contamination and those required to reduce the microbial load of fresh produce have a main impact on DBP formation.

In current literature, sanitizer efficacy is mainly expressed in terms of microbiological reductions, with much attention on produce decontamination and to a lesser extent on cross-contamination prevention. Nevertheless, one should also consider microbiological and chemical safety for the consumer as well as quality aspects such as the sensory and nutritional value of the produce when evaluating overall sanitizer efficacy. Many of these parameters can help to validate and weigh the usefulness of a sanitizer. Furthermore, a cost analysis of the implemented sanitizer could help to validate the economic cost efficacy of a fresh-cut produce treatment or investment for a certain disinfection technology. In addition, Gil, Selma, López-Gálvez and Allende [12] reference another pivotal influence, the variations between laboratory, pilot, and factory scale experiments with respect to sanitizer efficacy [10,33]. These differences may create another challenge when trying to evaluate sanitizer efficacy based on scientific literature or on a laboratory scale. 

## 4. Legislation and Disinfection By-Products 

In the European Union (EU), Regulation (EU) No 528/2012, applicable as of 1 September 2013, aims to improve the functioning of the internal market of biocidal products while ensuring a high level of environmental and human health protection [34]. With respect to this regulation, the European Chemicals Agency (ECHA) provides a summary of the status of applications in which substances including active chlorine (manufactured), ClO_2_, and PAA are currently under review; these substances are also recognized for review in Annex II Part 1 of the Commission Delegated Regulation (EU) No 1062/2014 of 4 August 2014 [34,35,36].

In the United States of America (USA), both the United States Environmental Protection Agency (EPA) and the United States Food and Drug Administration (FDA) exhibit judicial power regarding raw agricultural commodities that are washed in, for example, a fresh-cut facility. Sanitizers that are used for fresh produce are regulated as secondary direct food additives by the FDA, meaning they exhibit a technical effect during processing yet not in the finished product, although in some cases they are considered generally recognized as safe (GRAS). Disinfectants are registered as pesticides with the EPA [12]. 

The reaction of chemical disinfectants with water matrix constitutes leads to the production of DBPs. In particular, the challenges surrounding the presence of high amounts of organic matter and the resulting DBPs have raised scientific, industrial, and political concerns [12,13,27,37,38].

### 4.1. Chlorine

Several concerns arise as a result of the potential health and environmental concerns due to the formation of carcinogenic, halogenated DBPs such as trihalomethanes (THMs) and haloacetic acids (HAAs) during chlorine application [12,13,14,23,37,39]. Furthermore, the high or excessive use of chlorine, *i.e.* hyperchlorination, in order to combat the increasing organic load in the wash water may produce unacceptable levels of DBPs [23,39]. The potential formation of these toxic DBPs in addition to potential future regulatory restrictions has motivated scientists and processors to investigate alternative disinfection methods, such as ClO_2_, O_3_, and PAA during produce washing. Currently, there is lacking evidence on chlorine DBP residues in fresh produce (e.g., in prepared salads) and subsequent human exposure [40,41]. 

Some EU Member States have explicitly stated certain boundary conditions for chlorine use in fresh produce washing processes, e.g., the United Kingdom poses limits concerning free and total chlorine in the wash water, the pH, the produce residence time in the washing tank, and the produce to water ratio [42], whereas France mentions free chlorine residual limits in the wash water and limits the halogenated organic compounds on the produce [43]. During storage, the degradation of chlorine solutions leads to the formation of chlorate/perchlorate. These degradation products are introduced into the wash water during chlorination and it is undesirable to have these degradation products absorbed into the fresh produce. To avoid this, chlorine solutions should be stored in the dark, at cool temperatures, and in diluted solution if possible. Also, it is preferable to use sodium hypochlorite (NaClO) solutions within a few weeks after production. At 5 °C, the degradation is very limited in the absence of heavy metal contamination, and with an increase in temperature of 10 °C, the degradation rate increases 3–4 fold [44].

### 4.2. ClO_2_

The high oxidizing capacity of ClO_2_ can be attributed to its reactivity via oxidation rather than electrophilic or oxidation substitution as seen with chlorine [45]. Due to such selective mechanisms, ClO_2_ forms the major by-products chlorite and chlorate upon decomposition, yet no direct organochlorine compounds form [14,37,45,46]. When iodide is present in the water, more iodinated DBPs, especially iodoform, are formed with ClO_2_ than with chlorine, and iodinated DBPs may be more toxic than chlorinated DBPs [47]. Furthermore, since ClO_2_ is more selective and possesses a lower oxidation strength than chlorine, its reactivity is less sensitive to organic matter [13,45]. 

Within the EU, there are no regulations concerning ClO_2_ application in fresh-cut produce washing [39]. According to Regulation (EC) No 396/2005, the default maximum residue limit (MRL) for chlorate was 0.01 mg/kg [48]. However, the European Commission (EC) Standing Committee on the Food Chain and Animal Health (SCoFCAH) have recognized that such as default level does not cover the presence of chlorate due to legal uses of e.g., disinfectants and there are no indications of illegal use of chlorate as a pesticide [49,50]. Therefore, as a provisional solution, it was agreed that the individual member states will be given the ability to establish enforcement levels at the national level until risk management can take place based on European Food Safety Authority (EFSA) scientific opinion and monitoring data [50]. 

In the USA, the FDA permits ClO_2_ as an antimicrobial agent in the wash water of not raw agricultural commodities, provided that the residual ClO_2_ is below 3 ppm and the treatment is followed by a potable rinse or another specified preservative method [51].

### 4.3. O_3_

The main DBP of concern is bromate, which results from the oxidation of bromide to hypobromous acid. Hypobromous acid is further oxidized to bromite and bromate [52,53]. Bromide concentrations in natural waters are highly variable. However, in many water sources low concentrations of bromide (<20 µg/L) are present, yet are not considered problematic. Higher concentrations of bromide (50–100 µg/L) result in excessive bromate formation, and bromate becomes a serious problem when bromide levels exceed 100 µg/L [54,55]. Bromate induces deoxyribonucleic acid (DNA) damage and is a possible human carcinogen [54,55,56,57]. Bromate is particularly problematic because it is not biodegradable [57]. 

Bromo-organic DBPs have been identified and can form by the reaction of hypobromous acid with organic matter [58]. However, the concentrations of these bromo-organic compounds are usually far below current drinking water standards [55]. Non-brominated organic compounds result from the oxidative breakdown of organic matter. Alkenes, activated aromatic systems, amines, and sulfur-containing organic compounds can lead to the fast formation of low molecular weight organic compounds (e.g., organic acids, aldehydes, ketones, alcohols, and esters) [58,59,60]. The largest fraction of low molecular weight organic compounds constitutes organic acids, whereas aldehydes and ketones are only formed in small amounts [58]. Iodate is the main by-product formed by direct oxidations with molecular O_3_ in iodide containing waters. Iodate is considered non-problematic, because it is transformed back to iodide in the human body [55,59]. Amines are highly reactive towards O_3_, which leads to the formation of odorous agents (e.g., isovaleraldehydes, phenylacetaldehydes, isobutyraldehydes, and 2-methylbutyraldehyde). 

Within the EU and the USA, bromate presence is regulated in the drinking water with a maximum level of 10 µg bromate/L [61,62]. In the USA, O_3_ has been granted GRAS approval for direct contact with food products; it can be used as a sanitizer for foods when used at levels and by methods of application consistent with good manufacturing practices (GMP) [63]. O_3_ is classified by the FDA as a secondary direct food additive (processing aid) for foods, *i.a.* on raw agricultural products [64].

### 4.4. PAA

In comparison to the other process wash water disinfectants, PAA has the least potential of producing DBPs [14]. Unlike chlorine-based sanitizers, PAA degradation by-products can easily dissolve in water and are non-toxic; thus, making PAA an effective biocide [65,66]. Van Haute, Sampers, Jacxsens and Uyttendaele [14] have indicated that negligible or low levels of aldehydes may form. 

## 5. Process Wash Water Disinfectants 

### 5.1. Chlorine 

Chlorine application, for example as NaClO, calcium hypochlorite Ca(ClO)_2_ or chlorine gas (Cl_2_), is widely utilized due to its bactericidal properties and cost efficiency [2,6,13,14,37,38,67,68,69,70]. During produce washing, chlorine dissolves in the water causing HOCl, an efficient oxidizer for pathogen inactivation. However, HOCl can readily dissociate into hypochlorite ions (OCl-) at high pH or into Cl_2_ at low pH [37,38,68]. Typical industrial application of free chlorine concentrations range from 50 to 200 mg/L, with a short contact time (*i.e.* 1–2 min), and pH values between 6.0 and 7.5 in order to stabilize the HOCl form alongside minimizing corrosion of processing equipment [2,13,37,38,69,70]. 

Maintaining a stable HOCl form during washing remains a challenge since soil, debris, and exudates can accumulate and contribute to an increasing organic load [13,27,37,38,68]. Luo, *et al.* [71] have examined cross-contamination prevention during produce washing and specify that the free chlorine concentration (e.g., disinfectant residual) in the washing water is a main critical control factor for cross-contamination prevention [71,72]. During produce washing, an increasing organic load is evident from the increased COD and turbidity in the washing water, and declining disinfectant residual, which can be indirectly estimated by the oxidation reduction potential (ORP) [38,68]. In brief, the disinfectant residual and, if relevant, the pH of the process wash water are important to monitor *in situ*. 

Researchers have investigated the formation of DBPs during chlorine sanitization treatments for fresh-cut produce. For example, López-Gálvez, Allende, Truchado, Martínez-Sánchez, Tudela, Selma and Gil [39] found THM formation (217 ± 38 µg/L) in fresh-cut lettuce processing water following a 30 minute NaClO treatment (100 mg/L) in washing water with a COD of 700 mg/L in a reconditioning setup. THM formation on the fresh-cut lettuce was only detected under more extreme processing conditions with 60 min NaClO treatment (700 mg/L) in washing water with a COD of 1800 mg/L [39]. Hence, optimizing chlorine application and avoiding hyperchlorination during fresh-cut produce washing can help to avoid the excessive formation of THMs, while maintaining efficient microbial inactivation. 

In order to evaluate the effectiveness of chlorine application as a wash water disinfectant during fresh-cut lettuce processing, Van Haute, Sampers, Holvoet and Uyttendaele [3] examined the effects of the wash water quality during processing and chlorine treatment. These authors determined that maintaining a residual concentration of 1 mg/L free chlorine in the washing water during processing of lettuce leaves initially contaminated with *E. coli* O157 (*ca.* 4.0 log CFU/g) resulted in contaminations below 2.7 and 2.5 log CFU/100 mL, respectively for tap water and artificially processed water (CODs of 500 and 1000 mg O_2_/L were evaluated) [3]. Furthermore, the authors evaluated the total THM accumulation during wash water disinfection. THM levels reached 124.5 ± 13.4 µg/L following a 1 h washing processing with a COD of 1000 mg O_2_/L and a chlorine dose of 609.0 mg/L; however, THMs were not detected on the fresh-cut lettuce post rinse [3]. Overall, the authors stress dosage and the residual concentrations are key parameters to consider when evaluating the effectiveness of chorine as a wash water disinfectant [3]. In addition, these results provide evidence to apply lower chlorine concentrations; in order to avoid cross-contamination, online monitoring of chlorine levels during washing would be essential [3]. 

Optimizing the concentration of free chlorine during produce washing to ensure water quality and prevent cross-contamination in the processing water alongside the evaluation of DBPs that can form during this process presents a challenge for scientists and industry alike. Gómez-López, Lannoo, Gil and Allende [27] sought to evaluate these issues by simulating the fresh-cut processing of spinach inoculated with an *Escherichia coli* O157:H7 cocktail (5 log CFU/mL) treated with several concentrations of free chlorine. The authors concluded that maintaining a free chlorine residual at 7 mg/L during processing, attainable with continuous monitoring of the dose and adjusting the concentration applied as a result of increasing COD, is able to completely eliminate *E. coli* O157:H7 cells in the process wash water [27]. By simulating a pilot plant approach, these authors were able to provide evidence for minimizing chlorination levels that can be applied within industry; nevertheless, THMs (>1000 µg/L) were still generated and, consequently, these results support the overwhelming concern of DBP generation at higher chlorine doses that are commonly applied at industry [27]. 

Although chlorine is commonly applied as a disinfectant in processing wash water to prevent cross-contamination, recent studies are investigating alternatives disinfectants, hurdle technologies as well as chemical combinations in order to achieve a higher safety for fresh produce [67]. For example, in order to enhance chlorine efficacy on a pilot plant scale, the addition of the process aid T128—a chemical mixture that helps to stabilize HOCl in wash water with high organic matter—was examined [68]. T128 was determined to significantly reduce the occurrence and survival of *E. coli* O157:H7 in the wash water and cross-contamination to un-inoculated shredded iceberg lettuce. Hence, T128 application in chlorine fresh produce sanitization systems has the potential to increase safety margins during fresh-cut processing [68]. 

### 5.2. ClO_2_

ClO_2_ may be utilized as a gas or dissolved in water during produce processing [45,67]. Although gaseous ClO_2_ can reach and penetrate microorganisms better than aqueous sanitizers, industrial applications remain limited due to factors like worker’s safety and complications with on-site generation (instability) [13,45]. For example, when applying gaseous chemicals, like ClO_2_, or even Cl_2_ or O_3_, ambient concentration levels in the workplace should in principle be monitored to protect worker safety [44,73,74,75]. ClO_2_ has shown to effectively inactivate a broad range of microorganisms by disrupting membrane permeability hindering certain metabolic activities such as protein synthesis [37,45,67]. In particular, as a bactericide and virucide at lower concentrations (0.1 ppm), ClO_2_ is reported to be effective against several microorganisms, including: bacterial spores, amoebal cysts, Giardia cysts, *Cryptosporidium, Mycobacterium tuberculosis*, *Legionella, E. coli, Salmonella*, and *Listeria*. In addition, ClO_2_ has shown to affect the formation of biofilms by hindering re-growth [37,67].

ClO_2_ efficacy may be due to its high oxidation and penetration capacity that can function at a wide pH range. For example, with an increasing pH, the degree of inactivation is also reported to increase [13,37,45,67]. Such broad capacities make this disinfectant a promising choice within the fresh-cut produce industry. In particular, gaseous ClO_2_ has been reported to reach and penetrate microorganisms better than aqueous sanitizers [13,45]. In terms of more quality-related attributes, aqueous ClO_2_ has demonstrated variable results for spoilage micro flora in produce. Nevertheless, gaseous ClO_2_ does display acceptable findings. Gaseous ClO_2_ is promising in terms of prolonging shelf life storage; however, Gómez-López [45] notes that some authors have reported a bleaching effect due to gaseous treatments. On the other hand, sensory changes due to ClO_2_ gas treatments can vary depending on the product as well as applied concentrations and times. From these parameters, information on the effects of applying higher concentrations of gaseous ClO_2_ on the natural micro flora, which can protect produce from attachment from other microorganisms, is currently limited to date. 

Rodgers, *et al.* [76] has preliminarily investigated the efficacy of aqueous ClO_2_ (3 ppm and 5 ppm), as well as chlorine (100 and 200 ppm), O_3_ (3 ppm), and PAA (80 ppm), to determine reductions of *E. coli* O157:H7 and *L. monocytogenes* in an aqueous model system. Both pathogens decreased >5 log following a 2 to 5 min exposure, with ozone being most effective (15 s) followed by ClO_2_ (19 to 21 s), chlorinated trisodium phosphate (25 to 27 s) and PAA (70 to 75 s). In comparison, log reduction times (*i.e.* the time required to reduce populations 1 log at 21 to 23 °C) in the aqueous model system were significantly lower than those seen on the produce; this was partly due to the presence of organic matter on the produce surface [76]. More recently, López-Gálvez, Allende, Truchado, Martínez-Sánchez, Tudela, Selma and Gil [39] showed that aqueous ClO_2_ application (3 ppm) is effective in reducing pathogen cross-contamination in fresh-cut iceberg lettuce washing water, while also preventing THM formation [39,45]. In general, industrial scale experiments for aqueous ClO_2_ efficacy in fresh-cut produce washing with an emphasis on preventing re-contamination within the washing tank remain limited.

### 5.3. O_3_

O_3_ is a powerful oxidant (ORP = 2.07 V) in water treatment, second only to the hydroxyl radical (ORP = 2.80 V) [77]. It is produced commercially from pure oxygen or dry air by corona discharge of electricity (the most cost-effective method) or through photochemical reactions when low amounts are required (e.g., in laboratories). Due to the short half-life as well as the reactive and toxic nature, O_3_ has to be produced on-site [44,59,73,78]. Concentrations ≥30% in gas are unstable and can be explosive. Once produced, gaseous O_3_ is transferred into the water; however, O_3_ is not readily soluble in water [52,59,73,79]. Aqueous O_3_ reacts with (in) organic compounds via direct oxidation with molecular O_3_ or an indirect oxidation with hydroxyl radicals, formed from O_3_ decomposition [55,80]. Formation of the hydroxyl radical from O_3_ is promoted by increasing the pH (Reaction (1) and (2)), with the addition of H_2_O_2_, or by photolysis [56,81].

(1) O_3_ + OH^−^ ➔ O_2_ + HO_2_^−^

(2) O_3_ + HO_2_^−^ ➔ HO• + O_2_•^−^ + O_2_

O_3_ reacts primarily with activated aromatic structures, carbon–carbon double bonds, and non-protonated amines. Carbohydrates and fatty acids react slowly with O_3_, while amines, amino acids, nucleic acids, proteins, and protein functional groups react more rapidly. The reaction of O_3_ with inorganic compounds occurs mainly through the transfer of an oxygen atom to the inorganic compound in a two electron oxidation of said compound. The high reactivity of the hydroxyl radical leads to near diffusion controlled reaction rates with water matrix constituents [53]. Since O_3_ has a high reactivity towards (in) organic matter in the wash water, more O_3_ has to be dosed to maintain the residual compared to other process wash water disinfectants. 

Disinfection is generally more efficient at a slightly acidic pH [82,83,84] or relatively independent of pH in the range of pH 6 to 9, as has been observed for viruses, bacteria, and protozoa [73,85,86,87,88]. In addition, as hydroxyl ions initiate ozone decomposition, the ozone decay in water is higher at alkaline pH. Therefore, it is advised to perform washing at about pH 6 [14,53]. Besides the efficacy of O_3_ as a water disinfectant, the damage to fruit and vegetable tissues caused by contact with O_3_ should be critically considered. For various produce, including lettuce, apples, strawberries, blueberries, cantaloupes, and celery, no adverse effects on sensory quality were reported when washed in ozonated water in the range 2–10 mg/L for up to 5 min [63,76,89,90,91,92,93].

O_3_, as is ClO_2_, is easily removed from dilute aqueous solutions by turbulent aeration. Therefore, washing baths with turbulence created by water jets appear to be a better choice than baths where turbulence is generated by air nozzles. Due to its volatile nature, O_3_ is well-suited for use in plug flow reactors such as pipes (e.g., pipes used in fresh produce washing to assure a certain contact time with the disinfectant) [73]. In order to avoid O_3_ and ClO_2_ evaporation from washing tanks, especially at high residuals, covered tanks with limited headspace could be advantageous.

### 5.4. PAA

PAA (CH_3_CO_3_H) is the peroxide of acetic acid [94,95]. It is commercially available as a quaternary equilibrium mixture of acetic acid (CH_3_CO_2_H), H_2_O_2_, CH_3_CO_3_H, and H_2_O, as shown by Reaction (3) [66,95]. It is a colorless liquid with a piercing vinegar-like odor with a pH of less than 2 [94,95].

(3) CH_3_CO_2_H + H_2_O_2_ ↔ CH_3_CO_3_H + H_2_O

Vandekinderen, Devlieghere, De Meulenaer, Ragaert and Van Camp [66] elaborate on the antimicrobial properties of PAA as being related to the production of reactive oxygen species, which damage DNA and lipids, as well as cell membrane disruptions, blockage of enzymatic and transport systems, denaturation of proteins and enzymes, and increased cell membrane permeability via oxidation of sulfhydryl and disulfide bonds. These authors also note that commercially available PAA often contains considerable amounts of H_2_O_2_, which also possesses antimicrobial properties although such properties are predominated by PAA disinfection power [66]. In short, some limitations to the use of such a sanitizer include its instability at higher concentrations (15%); in particular, commercially available PAA solutions (10–15%) are more stable than other concentrations [95]. Other limitations include the higher costs (e.g., operational) in comparison to more traditional chlorine based methods [14,66]. However, its reported limited susceptibility to organic matter, broad temperature range usage, broad pH spectrum (3.0–7.5), and reported non-toxic decomposition products (being acetic acid and oxygen) make it a relevant alternative for application in the process wash water [66,96]. More recently though, Van Haute, López-Gálvez, Gómez-López, Eriksson, Devlieghere, Allende and Sampers [15] have found that the disinfection efficacy of PAA plus lactic acid showed increased efficiency with decreasing pH. PAA reacts more slowly with organic matter in the wash water than free chlorine, and as such a lower dose is necessary to maintain a desired residual [15,66]. However, the disinfection rate of PAA is also much slower, and as such a higher disinfectant residual is necessary to achieve equally rapid microbial inactivation [15,97]. 

Van Haute, Sampers, Jacxsens and Uyttendaele [14] also evaluated PAA based on managerial criteria such as costs and complexity of operation. In brief, PAA is reported to be more cost-effective on lower scale applications. In addition, inactivation of coliforms had required longer concentration and contact times for O_3_ in contrast to PAA or ClO_2_ [14]. Furthermore, Van Haute, Sampers, Jacxsens and Uyttendaele [14] stressed that PAA maintenance and operation, as also seen with hypochlorite solutions, are relatively simple to execute in comparison to other chemical disinfection methods.

Furthermore, the final quality related effects on the end product (e.g., post storage) should be considered. For example, Rodgers, Cash, Siddiq and Ryser [76] noted that sensory panelists were able to detect the use of 80 ppm of PAA on chopped lettuce. Unfortunately, such objectives are often not the main aim of studies; nevertheless, authors sometimes note sanitizer application in the process wash water as a final recommendation. For example, Baert, Vandekinderen, Devlieghere, Van, Debevere and Uyttendaele [31] highlight NaClO and PAA as useful sanitizers for cross-contamination prevention in the washing water as well as sanitizers that are necessary to maintain viral and bacterial microbiological quality of recycled wash water, yet these sanitizers were seen as less relevant for decontaminating microbial populations on the lettuce. 

In order to improve PAA efficacy, acquiring knowledge on the inactivation kinetics of microorganisms, including approaches that target multiple hurdle strategies such as combined physical and chemical or multiple chemical treatments, should be investigated. Sánchez, *et al.* [98] evaluated the applicability of PAA based sanitizer (Tsunami^®^ 100) in combination with high power ultrasound (HPU) to inactive the MNV-1 strain in the process wash water; however, these authors determined that these methods were insufficient during process washing since a rapid inactivation of MNV was necessary. MNV-1 was determined to be more resistant to hurdle technologies than pathogenic bacteria *E. coli* O157:H7 and *Salmonella*. In short, PAA efficacy was not enhanced when combined with HPU during MNV inactivation. Other HPU conditions like higher frequencies and combinations of HPU with physical treatments were recommended for further investigation. Nevertheless, these authors concluded that given the recommended concentrations of PAA, it is shown to be an alternative choice to chlorine-based sanitizers when preventing MNV cross-contamination during produce washing in the process wash water. Other PAA based sanitizers, such as a combination of lactic acid and peroxyacetic acid (LA-PAA) have been more recently investigated in terms of microbial efficacy and have been suggested as a possible alternative to chlorine based disinfectants in fresh produce washing water [99]. 

## 6. Conclusions

Various parameters can affect the efficacy of disinfection treatments on fresh-cut produce. Sanitizers should be used to maintain the quality of washing water in order to prevent cross-contamination rather than as a last resort for produce decontamination. The influence of organic matter on the disinfectant, and consequently the necessary residual concentration during process wash water disinfection, is critical to monitor, e.g., by online monitoring and dosing. In addition to the microbiological and the chemical safety of a disinfectant, the effect on product quality is essential to consider in parallel with legal aspects when selecting disinfectants and methods. Thus, optimizing the amount of sanitizer required for disinfection is key in order to reduce undesirable impacts, especially those that may negatively impact public health. Additional investigation into the influencing factors for the appropriate selection of disinfectants is critical. Further research on a sanitizer’s efficacy in the washing water is recommended at the laboratory scale, in particular with experimental designs reflecting industrial conditions. Validation on the industrial scale is warranted to better understand the overall effects of a sanitizer.
